# Insights into the species evolution of *Calanus* copepods in the northern seas revealed by *de novo* transcriptome sequencing

**DOI:** 10.1002/ece3.8606

**Published:** 2022-02-22

**Authors:** Apollo Marco Lizano, Irina Smolina, Marvin Choquet, Martina Kopp, Galice Hoarau

**Affiliations:** ^1^ Faculty of Biosciences and Aquaculture Nord University Bodø Norway; ^2^ Department of Medical Biochemistry and Microbiology Uppsala University Uppsala Sweden

**Keywords:** *Calanus*, concordance factor, de novo transcriptome, phylotranscriptomics, RNA‐seq

## Abstract

Copepods of the zooplankton genus *Calanus* play a key role in marine ecosystems in the northern seas. Although being among the most studied organisms on Earth, due to their ecological importance, genomic resources for *Calanus* spp. remain scarce, mostly due to their large genome size (from 6 to 12 Gbps). As an alternative to whole‐genome sequencing in *Calanus* spp., we sequenced and de novo assembled transcriptomes of five *Calanus* species: *Calanus glacialis*, *C. hyperboreus*, *C. marshallae*, *C. pacificus*, and *C*. *helgolandicus*. Functional assignment of protein families based on clusters of orthologous genes (COG) and gene ontology (GO) annotations showed analogous patterns of protein functions across species. Phylogenetic analyses using maximum likelihood (ML) of 191 protein‐coding genes mined from RNA‐seq data fully resolved evolutionary relationships among seven *Calanus* species investigated (five species sequenced for this study and two species with published datasets), with gene and site concordance factors showing that 109 out of 191 protein‐coding genes support a separation between three groups: the *C*. *finmarchicus* group (including *C*. *finmarchicus*, *C. glacialis*, and *C. marshallae*), the *C*. *helgolandicus* group (including *C*. *helgolandicus*, *C. sinicus*, and *C*. *pacificus*) and the monophyletic *C. hyperboreus* group. The tree topology obtained in ML analyses was similar to a previously proposed phylogeny based on morphological criteria and cleared certain ambiguities from past studies on evolutionary relationships among *Calanus* species.

## Graphical abstract

This study contributes and improve the currently available transcriptomic resources for *Calanus* spp. and explore de novo transcriptome data as an alternative to whole genome sequencing to infer evolutionary relationships within the genus *Calanus*. Here, we sequenced, assembled, and annotated de novo transcriptomes of two species for the first time (*C. hyperboreus* and *C. marshallae*), and three species with some but limited transcriptomic data available (*C*. *glacialis*, *C*. *helgolandicus*, and *C. pacificus*).

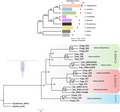

## 1 INTRODUCTION

Recent developments of next‐generation sequencing (NGS) technologies for nucleotide sequencing have revolutionized the field of molecular biology (Metzker, 2010; Schuster, 2008) by allowing the generation of massive amounts of data more rapidly and cost‐efficiently than ever before (Luikart et al., 2003). Nonetheless, generating whole‐genome data can still be challenging for many zooplankton groups, due to their typically small body size yielding only small amounts of DNA, usually not enough for whole‐genome sequencing, exacerbated by their often large and complex genome architecture characterized by the presence of many repetitive sequences (Bucklin et al., 2018; Tarrant et al., 2019). Copepod species of the marine zooplankton genus *Calanus*, although morphologically very similar (Choquet et al., 2018; Fleminger & Hulsemann, 1977; Frost, 1974), have large genomes that differ greatly in size (from nearly 6 Gbps in *C*. *finmarchicus* to 12 Gbps in *C. hyperboreus*, McLaren et al., 1988). *Calanus* species play a key role in energy transfer in marine food webs, both as primary consumers and as prey for fish, seabirds, and other marine predators (Arnkværn et al., 2005; Bonnet & Frid, 2004; Cleary et al., 2017). Despite their ecological importance, genomic resources currently available for *Calanus* spp. remain limited, which has led to poor understanding of phylogenetic relationships within the *Calanus* genus so far.

Before development of genetic tools, a phylogeny based on the analysis of morphological characteristics (i.e., relative size of accessory photoreceptor, caudal ramus, anal segment, and genital pore—see Frost, 1974) was proposed for species of the genus *Calanus* (Frost, 1974 and reported in Bucklin et al., 1995). The morphology‐based phylogeny identified two distinct groups: the *C*. *finmarchicus* group, including *C*. *finmarchicus*, *C. glacialis*, and *C*. *marshallae*; and the *C*. *helgolandicus* group, including *C*. *helgolandicus*, *C*. *pacificus*, *C. sinicus*, and four other species not investigated in the present study. *Calanus hyperboreus* was considered as a separate clade, distinct from the *C*. *finmarchicus* and *C*. *helgolandicus* groups. Later, new phylogenies emerged from analyses of the two genetic markers 16S rRNA (Bucklin et al., 1995) and 28S rRNA (Kozol et al., 2012), but showed a lack of congruence. Although the 28S‐based phylogeny proposed by Kozol et al. (2012) seemed to agree with the morphology‐based phylogeny on the clustering of species, some branches were not well supported. In contrast, the 16S‐based phylogeny suggested a different grouping of species, with several species from the “*C*. *helgoIandicus* group” identified by Frost (1974) not clustering together (i.e., *C*. *pacificus* separated from *C*. *sinicus* and *C*. *helgolandicus*; Bucklin et al., 1995). The discrepancy observed between the two different molecular phylogenies may be explained by the potentially limited resolution of using only a single molecular marker. The use of NGS approaches to obtain larger numbers of molecular markers from genome‐wide data can overcome this problem and provide more powerful datasets, needed to accurately characterize species relationships (Leaché & Oaks, 2017).

There has been a growing interest in utilizing RNA‐seq approaches to answer evolutionary questions for nonmodel species because of the ease in assembling and analyzing transcriptome data compared to genomic data and of the possibility to obtain additional information from exonic regions of multiple genes (see e.g., Bi et al., 2012; Tarrant et al., 2019). Transcriptomics studies are on the rise in nonmodel marine organisms (Eldem et al., 2017; Marlétaz et al., 2019; Pai et al., 2018; Tarrant et al., 2019; Ungaro et al., 2017), but are still too limited to understand zooplankton species ecology and evolution (Lenz et al., 2021). For the genus *Calanus*, various RNA‐seq studies have investigated, for example, the classification of genes associated with developmental cycles from embryos to adult (Lenz et al., 2014) and genes contributing to molecular mechanisms during diapause, diapause termination, and starvation (Ohnishi et al., 2019; Skottene et al., 2019). In addition, studies have also looked at patterns of daily gene expression changes at different latitudes and sea‐ice coverage (Payton et al., 2020) and the effects of ocean acidification on the regulation of gene expression (Bailey et al., 2017). Yet, there have been no studies utilizing RNA‐seq‐based data to mine genes to resolve the phylogeny of the genus *Calanus*. Recent studies have validated the use of transcriptomes in phylogenetic analyses, showing virtually identical results with phylogenies derived from whole or partial genome, regardless of the tissue origin and whether the same tissue was used across species (Cheon et al., 2020; Zhao et al., 2021). Multiple studies have already used transcriptomics for phylogenetics of various marine organisms, including dinoflagellates (Annenkova et al., 2018), pteropods (Peijnenburg et al., 2020), bivalves (Li et al., 2020), and crustaceans (Gan et al., 2020).

For the genus *Calanus*, there are currently 17 independent RNA‐seq datasets available in the NCBI SRA database (Appendix Table S1, accessed November 2020) representing five *Calanus* species (*C. helgolandicus*, *C. finmarchicus*, *C. sinicus*, *C. pacificus*, and *C. glacialis*), of which only three species have a complete transcriptome assembly: *C*. *helgolandicus* (Asai et al., 2020), *C*. *finmarchicus* (Lenz et al., 2014; Tarrant et al., 2014); and *C*. *sinicus* (Ning et al., 2013; Yang et al., 2014). For *Calanus* species living in the northern seas (covered by the North Atlantic, Arctic, and North Pacific oceans), where they dominate the zooplankton biomass, RNA‐seq studies have targeted mostly four species (i.e., *Calanus helgolandicus*, *C. finmarchicus*, *C. sinicus*, and *C. glacialis*), while other species have been mostly ignored (*C. pacificus*, *C. marshallae*, and *C. hyperboreus*).

Our objective is to contribute and improve the currently available transcriptomic resources for *Calanus* spp. and explore the suitability of de novo transcriptome data to infer evolutionary relationships within the genus *Calanus*. To achieve this, we sequenced, assembled, and annotated de novo transcriptomes of two *Calanus* species for the first time (*C. hyperboreus* and *C*. *marshallae*) and of three species with limited transcriptomic data available (*C*. *glacialis*, *C. helgolandicus*, and *C. pacificus*). We also investigated hundreds of single‐copy orthologs present among the seven species of *Calanus* derived from RNA‐seq data and updated the phylogeny of the genus *Calanus*.

## 2 MATERIALS AND METHODS

### 2.1 Specimen collection and molecular species identification

**FIGURE 1:**
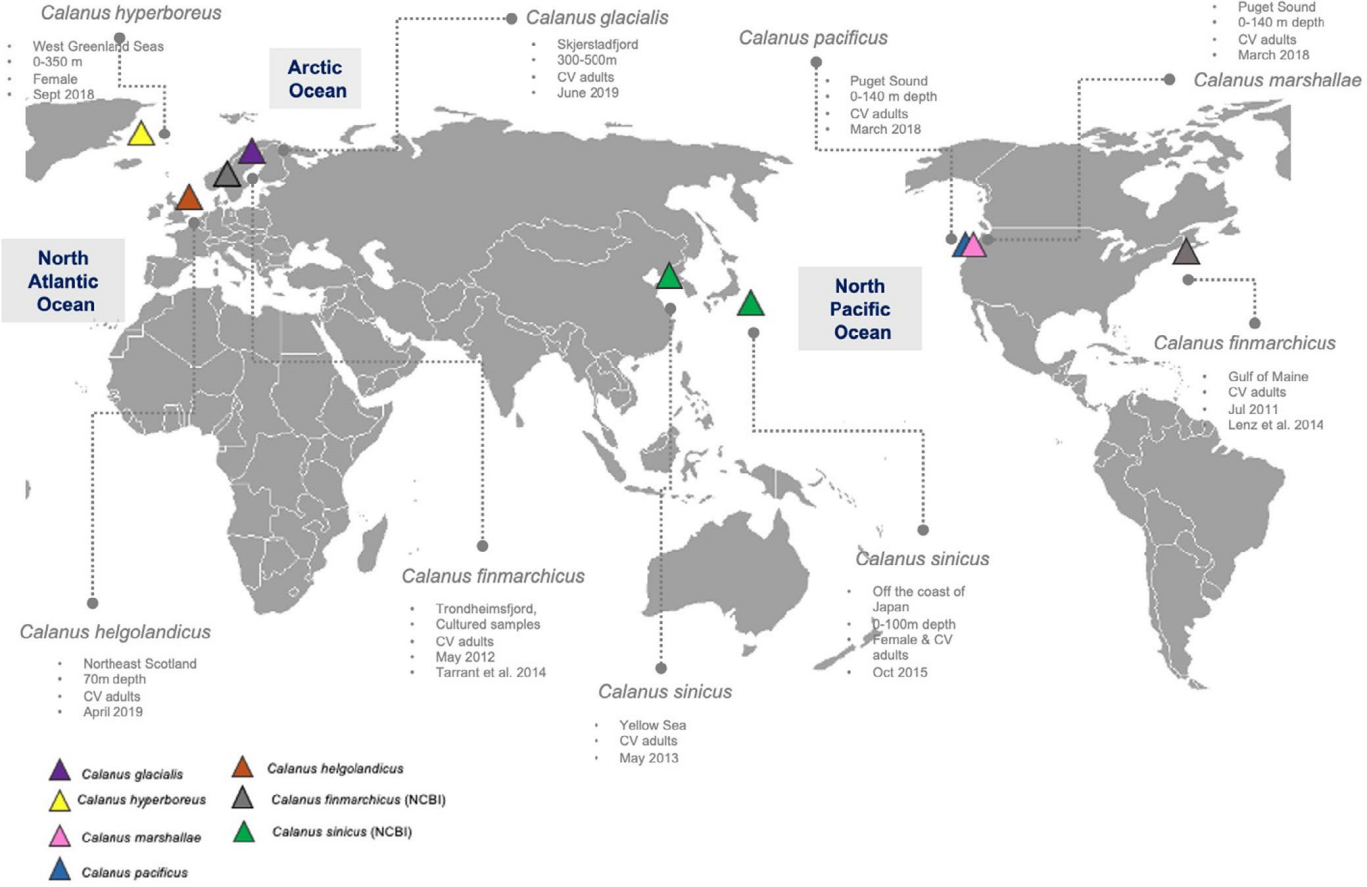
Sampling locations for the seven species of *Calanus* analyzed in this study

Three individual copepodites of five species of *Calanus* (*C. helgolandicus*, *C. pacificus*, *C. glacialis*, *C. marshallae*, and *C. hyperboreus*) were sourced from various collaborators (see details in Table 1). These specimens originated from zooplankton samples collected between March 2018 and June 2019 at different sites across the North Atlantic, the North Pacific, and the Arctic Oceans (Figure 1), from different depth ranges (Table 1) by vertically towing a plankton net (WP3, Juday or multinet). For each sample, *Calanus* spp. individuals were pre‐sorted from the rest of the zooplankton and subsequently preserved at −20°C in RNAlater. Individuals of *C*. *pacificus* and *C*. *marshallae* were morphologically identified as such by the taxonomist B. Frost. Molecular species identification was performed to confirm the species identity of *C*. *helgolandicus*, *C. glacialis*, and *C. hyperboreus* using six InDel (Insertion‐Deletion) molecular markers (Smolina et al., 2014), with DNA extracted from antennules separately, following the optimized protocol from Choquet et al. (2017).

**TABLE 1:** Sampling information for *Calanus* species from the North Atlantic, Arctic, and North Pacific Oceans used in this study

Species	Individual ID	Date of collection	Sampling site	Coordinates		Sampling depth (m)	Developmental stage	Collector or study
				Lat	Lon			
*Calanus glacialis*	Cgla_007	06/2019	Skjerstadfjord	67°14′N	14°44′E	300–500	CV	M. Krogstad
	Cgla_010							
	Cgla_011							
*Calanus hyperboreus*	Chype_012	09/2018	West Greenland Sea	74°34′N	11°18′W	0–350	Adult female	E. Friis Møller
	Chype_021							
	Chype_030							
*Calanus marshallae*	Cmar_005	03/2018	Main basin of Puget Sound	47°40′N	122°28′W	0–140	CV	A. Bucklin & B. Frost
	Cmar_007							
	Cmar_008							
*Calanus pacificus*	Cpac_006	03/2018	Main basin of Puget Sound	47°40′N	122°28′W	0–140	CV	A. Bucklin & B. Frost
	Cpac_007							
	Cpac_008							
*Calanus helgolandicus*	Chelg_003	04/2019	Stonehaven ‐ north‐east Scotland	56°57′N	02°07′W	0–48	CV	L. Noble
	Chelg_007							
	Chelg_008							
*Calanus finmarchicus*	Cfin_SRR1153468	07/2011	Mount Desert Rock, Gulf of Maine	44°2′N	68°3′W	Not specified	CV	Lenz et al. (2014)
	Cfin_SRR1141107	05/2012	NTNU/SINTEF Sealab facility Trondheim, Norway	Not specified	Not specified	70		Tarrant et al. (2014)
	Cfin_SRR1141110			Not specified	Not specified			
*Calanus sinicus*	Csin_DRR144876	10/2015	Off the coast of Japan along the Kuroshio Current	34°00′N	138°00′E	0–100	Adult female	Ohnishi et al. (2019)
	Csin_DRR144878							
	Csin_SRP032493	05/2013	Yellow Sea	38°45′N	121°45′E	Not specified	Adult copepod unspecified sex	Yang et al. (2014)
*Acartia tonsa*	Atonsa_Nilsson	09/2016	Øresund Denmark	56°N	12°E	Culture	Adult	Nilsson et al. (2018)
*Eurytemora affinis*	Eurytemora_affinis	NA	Bred at WHOI for 1 year	NA	NA	Culture	Adult female	Almada & Tarrant (2016)

Note

Three individuals were used for each species. For *C*. *finmarchicus* and *C*. *sinicus*, previously published data were used, individual ID contains the reference number for sequences downloaded from the NCBI SRA database.

### 2.2 RNA extraction, library preparation, and sequencing

Total RNA was extracted from the 15 pre‐identified (using antennules DNA or morphology) *Calanus* individuals using the Tri(Qia)zol from Qiagen RNeasy mini kit with minor modifications to the manufacturer's protocol and was concentrated using the RNA Clean & Concentrator™ kit (Zymo Research). RNA quality was assessed using an Agilent Bioanalyzer 2100 (Agilent Technologies) and showed integrity numbers (RIN values) between 9.6 and 10.0, indicating high quality of extracted RNA. Aside from RIN values, we did not observe evidence of DNA contamination and limited smearing in small size range.

Individual RNA libraries were prepared using the NEBNext^®^ Ultra™ II Directional RNA Library Prep Kit for Illumina^®^ (New England Biolabs) following the manufacturer’s protocol. Libraries were quantified using an Agilent Bioanalyzer 2100 (Agilent Technologies) and pooled in equimolar concentrations before sequencing on an Illumina NextSeq 500 platform with a 2 × 150 bp high‐output NextSeq 500/550 v. 2.5. kit.

### 2.3 De novo transcriptome assembly and transcript filtering

Raw sequencing reads, generated from the 15 individuals, were de‐multiplexed using bcl2fastq v. 2.2 (https://support.illumina.com/sequencing/sequencing_software/bcl2fastq‐conversion‐software.html) and quality checked using FastQC v. 0.11.5 (http://www.bioinformatics.babraham.ac.uk/projects/fastqc/). Low‐quality reads (see Appendix S1) and adapter sequences were trimmed using cutadapt v. 1.18 (Martin, 2011).

RNA‐seq reads from transcriptomes of two additional *Calanus* species (*C*. *finmarchicus* and *C*. *sinicus*) with three individuals per species, and two closely related taxa (*Acartia tonsa* and *Eurytemora affinis*) with one individual per species, were downloaded from the NCBI SRA database and included in the subsequent analyses (see Table 1 for more information). We chose to sequence new individuals for both *C*. *glacialis* and *C*. *helgolandicus* as most of the transcriptome assemblies available online are either incomplete or limited to certain species‐specific stages. In order to compare across species, we aimed for only CV or adult females. We selected *Acartia tonsa* and *E*. *affinis* as outgroups since they are among the closest extant species to *Calanus* spp. within the order Calanoida to have complete transcriptomes available online (Tarrant et al., 2019), and importantly because both species have been used as outgroups in a previous study where divergence time was estimated (Eyun, 2017).

Individual de novo transcriptome assemblies were performed for all individuals of seven species using Trinity v. 2.9.1. (Grabherr et al., 2011). Assembly statistics were computed using the Perl script TrinityStats.pl contained in the Trinity software package. Based on the lengths of assembled transcriptome contigs, we computed for N50 based on the single longest isoform per gene (Nx50) and ExN50 statistics, which are limited to the topmost highly expressed genes. N50 values can often be exaggerated due to Trinity program generating too many transcript isoforms. To attenuate the probability of including false and redundant transcripts, contigs were filtered in five steps: (1) cross‐species contamination of contigs in assembled transcriptome was removed using CroCo v. 1.1 (Simion et al., 2018); (2) weakly expressed transcript isoforms or isoforms which are not expressed as much as other isoforms were removed and only the most highly expressed isoform per gene were retained using Trinity perl scripts *align_and_estimate_abundance*.*pl* and *filter_low_expr_transcripts*.*pl* with the “‐‐highest_iso_only” parameter included; (3) redundant transcripts with ≥95% identity were filtered out using cd‐hit‐est v. 4.7 (Fu et al., 2012; Li & Godzik, 2006); (4) misassembled or incomplete contigs were filtered out based on read mapping metrics using TransRate v. 1.0.3 (Smith‐Unna et al., 2016); and lastly, (5) only transcripts containing open‐reading frames (ORFs) with a length of at least 100 amino acids were retained using Transdecoder v. 5.5 (Haas et al., 2013, Figure S1). Then, transcriptome assembly completeness was assessed using BUSCO v. 4.0.2 (*Benchmarking Universal Single*‐*Copy Orthologs*, Seppey et al., 2019) to obtain the overview of all single‐copy, duplicated, and missing orthologs represented in the arthropod dataset (arthropoda_odb10). Downstream analyses were performed using the resulting 23 filtered transcriptomes.

### 2.4 Identification of coding regions and functional annotation

Candidate coding regions within the transcriptome assemblies were identified using TransDecoder v. 5.5 (Haas et al., 2013). Functional annotation was performed using eggNOG‐mapper v. 2.0 (Huerta‐cepas et al., 2017) based on fast orthology assignment using precomputed eggNOG v. 5.0 (Huerta‐Cepas et al., 2019) clusters and phylogenies. Protein families were assigned to known functional class using gene ontology (GO) terms and the database of clusters of orthologous genes (COG, Galperin et al., 2021). Two COG groups “Cell motility, N” and “Nuclear Structure, Y” showed very low protein counts across the seven *Calanus* species, and the reason behind it was investigated in detail using eggNOG database for Arthropoda (6656 single‐copy orthologs, downloaded on 25.04.2021). Proteins in the Arthropoda database for N and Y categories that were not identified by eggNOG‐mapper in *C. hyperboreus* (species with the lowest number of obtained matches to N and Y category) were manually searched using HHMER v3.1b2 (Johnson et al., 2010). The created hidden Markov models were used to mine predicted ORFs from *C. hyperboreus*. Best significant hits (with the lowest *E*‐value) were searched against NCBI nonredundant protein database using BLASTp to further confirm their identity and relatedness to COG functional categories. In addition, annotated transcripts were also classified to three GOSlim functional categories (biological process, cellular component, and molecular function) using the webserver “PANTHER Classification System” (Mi et al., 2019, 2021) with *Drosophila melanogaster* chosen as the reference organism.

### 2.5 Ortholog identification and phylogenetic analyses

Orthofinder v. 2.3.1 (Emms & Kelly, 2019) was used to prepare a dataset containing only single‐copy orthologs for phylogenetic inference using all 23 transcriptomes, while for estimation of gene duplication events along the phylogeny only representative individuals per species were used. A phylogenetic tree was constructed by aligning protein‐coding sequences of single‐copy orthologs using clustalomega v. 1.2.4 (Sievers & Higgins, 2014). A customized Python script (*convert*.*py*, https://github.com/mmatschiner/tutorials/tree/master/ml_species_tree_inference) was used to remove sequences containing missing information from all the alignments and to translate each sequence alignment into a Nexus format, needed for IQ‐tree. Maximum likelihood (ML) phylogenetic trees were generated for each single‐copy orthogroup using both the bootstrap method (with 1000 replicates) and the maximum‐likelihood method with branch lengths calculation using IQ‐tree ver. 1.6.1 (Nguyen et al., 2015). Substitution models were not specified, allowing IQ‐tree to choose the best‐fitting model for each orthogroup. A single species tree with maximum number of quartets shared among gene trees based on ML was inferred among individual species trees using ASTRAL v. 5.7.3 (Zhang et al., 2018). Gene and site concordance factors (sCF) were also computed to determine which branches show concordant and disconcordant genes and to calculate site variances to the reference ML tree.

## 3 RESULTS

### 3.1 Sequencing

We sequenced 15 individual transcriptomes, with three individuals for each of five *Calanus* species (*C. helgolandicus*, *C. pacificus*, *C. glacialis*, *C. marshallae*, and *C. hyperboreus*). The mean sequencing output per individual was 47.6 million reads (ranging from 10.8 to 102 million, Table S2). All raw reads were uploaded to NCBI SRA database under BioProject PRJNA744376 and all de novo transcriptome assemblies generated in this project has been deposited at DDBJ/EMBL/GenBank TSA database and DRYAD server (https://doi.org/10.5061/dryad.n8pk0p2ww, see Table S4).

### 3.2 De novo transcriptome assembly and quality assessment

De novo transcriptome assembly using Trinity was performed for each *Calanus* individual sequenced in addition to six datasets publicly available, generating a total of 21 transcriptome assemblies. Quality metrics for each assembly are presented in Table 2. Mapping of quality trimmed reads to their corresponding de novo transcriptome assemblies generated alignment rates from 95.62% to 99.13% with a mean alignment rate of 96.69%. Based on 1013 conserved arthropod orthologs, our BUSCO analysis identified 93.57% (mean among three individuals) complete single‐copy and complete duplicated BUSCO’s in *C. glacialis*, (91.33%) in *C. hyperboreus*, (88.43%) in *C*. *marshallae*, (91.80%) in *C. pacificus*, and (92.10%) in *C*. *helgolandicus*. These parameters indicated that the 15 de novo transcriptomes were well assembled and relatively complete (Table 2; Figure S2). Moreover, we investigated orthologs identified by BUSCO as missing and found eight orthologs that are common among all the 21 *Calanus* transcriptomes (Table S3). However, further manual BLAST of these proteins against the translated transcriptome of *C. hyperboreus* revealed query hits >50% for six proteins, and the absence of significant hits for the two other proteins: 3‐ketodihydrosphingosine reductase (*Plutella xylostella*, XP_037969682.1) and serine palmitoyltransferase 1 (*Plutella xylostella*, XP_037971886.1), both reportedly involved in sphingolipid metabolism.

**TABLE 2:** Transcriptome assembly statistics for *Calanus* spp. and two outgroup species *Acartia tonsa* and *Eurytemora affinis* downloaded from NCBI SRA database showing the total no. of assembled bases, total no. of genes, total no. of transcripts, %GC content, % alignment, no. of retained transcripts, no. of peptides (ORF ≥ 100 aa), and BUSCO results

Species	Individual ID	Total no of assembled bases	Total no genes	Total no of transcripts	%GC	% alignment	No of retained transcripts	No of peptides (OR*F* ≥ 100 aa)	BUSCO
*Calanus glacialis*	Cgla_007	80,786,787	107,689	191,809	42.61	98.10	58,057	55,050	C: 93.7% [S: 53.0%, D: 40.7%], F: 1.2%, M: 5.1%
	Cgla_010	52,916,092	107,265	191,130	42.63	95.64	82,924	69,473	C: 93.8% [S: 51.8%, D: 42.0%], F: 0.8%, M: 5.4%
	Cgla_011	83,871,692	117,560	208,862	42.63	95.62	41,072	72,199	C: 93.2% [S: 50.1%, D: 43.1%], F: 1.4%, M: 5.4%
*Calanus hyperboreus*	Chype_012	64,862,613	89,686	154,261	43.86	99.13	57,073	43,074	C: 92.0% [S: 48.7%, D: 43.3%], F: 1.8%, M: 6.2%
	Chype_021	44,640,167	57,792	98,478	44.80	96.87	55,106	67,106	C: 89.6% [S: 53.0%, D: 36.6%], F: 2.5%, M: 7.9%
	Chype_030	69,174,829	97,334	165,321	43.13	96.05	79,215	64,262	C: 92.4% [S: 51.3%, D: 41.1%], F: 1.6%, M: 6.0%
*Calanus marshallae*	Cmar_005	43,950,718	58,868	86,851	45.35	96.61	30,505	34,572	C: 88.8% [S: 59.5%, D: 29.3%], F: 1.8%, M: 9.4%
	Cmar_007	53,405,794	70,397	108,982	44.96	97.07	42,388	46,829	C: 89.8% [S: 56.4%, D: 33.4%], F: 2.2%, M: 8.0%
	Cmar_008	30,405,070	39,378	55,916	45,89	95.86	48,111	53,770	C: 86.7% [S: 58.7%, D: 28.0%], F: 2.1%, M: 11.2%
*Calanus pacificus*	Cpac_006	57,260,966	79,107	133,448	45.60	96.53	57,755	60,389	C: 90.7% [S: 38.2%, D: 52.5%], F: 2.0%, M: 7.3%
	Cpac_007	65,331,309	85,103	153,092	45.30	96.68	48,704	56,214	C: 93.5% [S: 40.4%, D: 53.1%], F: 1.8%, M: 4.7%
	Cpac_008	62,163,761	87,403	144,545	45.31	96.34	76,121	72,916	C: 91.2% [S: 38.4%, D: 52.8%], F: 2.5%, M: 6.3%
*Calanus helgolandicus*	Chelg_003	82,334,519	110,120	199,181	44.70	97.17	62,642	67,106	C: 93.6% [S: 39.8%, D: 53.8%], F: 1.2%, M: 5.2%
	Chelg_007	61,489,417	83,960	137,554	45.23	96.31	54,695	60,542	C: 90.6% [S: 41.6%, D: 49.0%], F: 2.2%, M: 7.2%
	Chelg_008	45,658,464	61,171	96,333	45.86	96.34	44,779	49,783	C: 89.3% [S: 45.3%, D: 44.0%], F: 2.9%, M: 7.8%
*Calanus finmarchicus*	Cfin_SRR1141107	29,971,015	43,252	75,504	46,28	88.89	53,751	51,970	C: 81.3% [S: 42.2%, D: 39.1%], F: 8.1%, M: 10.6%
	Cfin_SRR1141110	39,691,175	53,703	113,701	45,39	88.73	25,298	33,135	C: 86.7% [S: 27.8%, D: 58.9%], F: 4.7%, M: 8.6%
	Cfin_SRR1153468	62,399,753	90,151	229,051	44,22	96.20	34,899	43,427	C: 90.2% [S: 54.5%, D: 35.7%], F: 4.3%, M: 5.5%
*Calanus sinicus*	Csin_SRP032493	61,756,777	102,986	235,405	46,44	94.98	67,651	61,031	C: 92.1% [S: 20.2%, D: 71.9%], F: 2.1%, M: 5.8%
	Csin_DRR144876	63,116,211	113,366	282,710	46,23	94.98	77,949	63,851	C: 91.8% [S: 11.5%, D: 80.3%], F: 2.5%, M: 5.7%
	Csin_DRR144878	58,403,986	106,792	263,104	46,16	94.67	69,934	58,166	C: 90.4% [S: 10.4%, D: 80.0%], F: 2.6%, M: 7.0%
*Acartia tonsa*	Atonsa_Nilsson	118,203,047	48,149	114,717	37,9	98.79	31,986	16,174	C: 56.8% [S: 56.2%, D: 0.6%], F: 2.8%, M: 40.4%
*Eureytemora affinis*	Eaffinis_Almada	181,412,865	90,855	170,681	38,61	95.23	57,397	24,056	C: 71.1% [S: 69.4%, D: 1.7%], F: 2.7%, M: 26.2%

Note

BUSCO assessment was based on arthropoda database (*odb_10* containing 1103 orthologs). C = complete, S = single, D = duplicated, F = fragmented, and M = missing no. of orthologs.

We tested for cross‐species contamination among the 15 new transcriptomes that were sequenced on the same flow‐cell, and none was detected. After filtering out weakly expressed isoforms, a total of 332,489 transcripts (representing 66.44% of all the generated transcripts) were retained for all individuals of *C. glacialis*, 58.56% for *C. hyperboreus*, 66.99% for *C*. *marshallae*, 58.37% for *C. pacificus*, 58.94% for *C. helgolandicus*, 44.73% for *C*. *finmarchicus*, and 43.75% for *C*. *sinicus*. The final number of peptides with ORF meeting the minimum criteria set by Transdecoder‐v.5.5 (Haas et al., 2013) ranged from 33,135 peptide sequences for *C. finmarchicus_SRR1141110* to 72,916 for *C. pacificus_008*. The mean number of remaining peptides among the seven species was 53,265 (Table 2).

### 3.3 Functional classification of protein families

**FIGURE 2:**
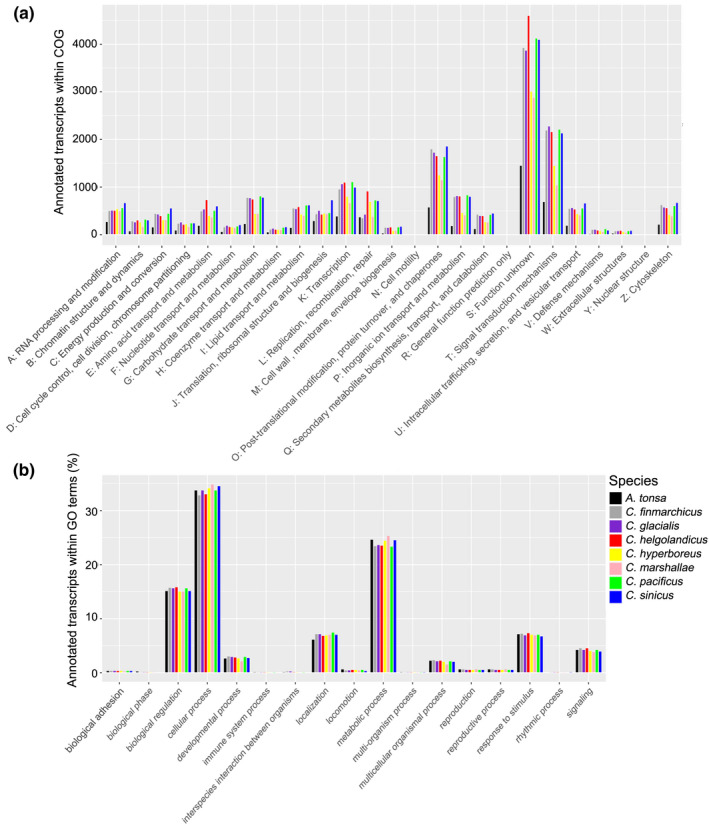
(a) Functional annotation of protein‐coding sequences based on Clusters of Orthologous Groups (COG) database. (b) Gene Ontology (GO) annotation representing biological process for seven species of *Calanus* and one outgroup taxon *Acartia tonsa*

Functional annotation of orthologous protein families was based on GO terms and COG databases implemented in eggNOG‐mapper v.2.0 (Huerta‐Cepas et al., 2017). Functional classification of protein‐coding sequences based on COG yielded different numbers of protein queries from one species to another. For *A*. *tonsa*, we were able to functionally assign 5947 protein queries, 10,925 *for C*. *marshallae*, 12,435 for *C. hyperboreus*, 16,057 for *C*. *finmarchicus*, 16,308 for *C*. *glacialis*, 16,971 for *C. pacificus*, 17,349 for *C*. *helgolandicus*, and 17,706 for *C*. *sinicus*. The orthologous protein families were subdivided into 25 COG classifications (Figure 2a). Among them, the category “Unknown Function, S” represented the largest group with a cumulative query hit comprising of 24.62% of the total protein assignments for all the *Calanus* species. It was followed by “Signal transduction mechanisms, T” (12.48%), “Post‐translational modification, protein turn‐over, and chaperon, O” (10.27%). Groups with the lowest protein count were linked to functions related to “Cell motility, N” (0.05%), “Nuclear Structure, Y” (0.04%), and “General function prediction only, R” (0,0%). In‐depth look into N and Y categories using Arthropoda eggNOG database showed that this database only contains 15 and 10 proteins in N and Y categories respectively, thus explaining general low numbers of these categories in *Calanus* transcriptomes (from 5 to 10 proteins per species in N category and from 5 to 8 proteins per species in Y category). Furthermore, manual search with HHMER reduced the number of unfound proteins from the Arthropoda database in *C. hyperboreus* from 10 to 5 in N category, and from 5 to 1 in Y category.

In total, the number of transcripts with GO and KEGG annotations were, respectively, 4161 and 4447 for *A*. *tonsa*; 10,869 and 12,106 for *C*. *finmarchicus*; 10,837 and 12,197 for *C*. *glacialis*; 7701 and 8305 for *C*. *marshallae*; 11,027 and 11,908 for *C*. *helgolandicus*; 11,231 and 12,313 for *C*. *pacificus*; 12,154 and 13,271 for *C*. *sinicus*; and 8575 and 9220 for *C. hyperboreus*. Overall, the percentage of annotated transcripts to major GO categories was similar, if not comparable among all examined species (Figure 2b). Within the GO category Biological Process (BP), the largest proportion of transcripts were assigned to cellular process (~33%), metabolic process (~24%), and biological regulation (~15%). Within the GO category Molecular Function (MF), binding and catalytic activities were the largest terms with ~40% and ~34%, respectively, of all transcripts with GO term hits. For Cellular Component (CC), within GO_Slim category in Panther database, transcripts were assigned to only three groups with cellular anatomical entity (~42%) and intracellular (~37%) as the largest groups (Figure S3).

### 3.4 Ortholog identification and phylogenetic analyses

**FIGURE 3:**
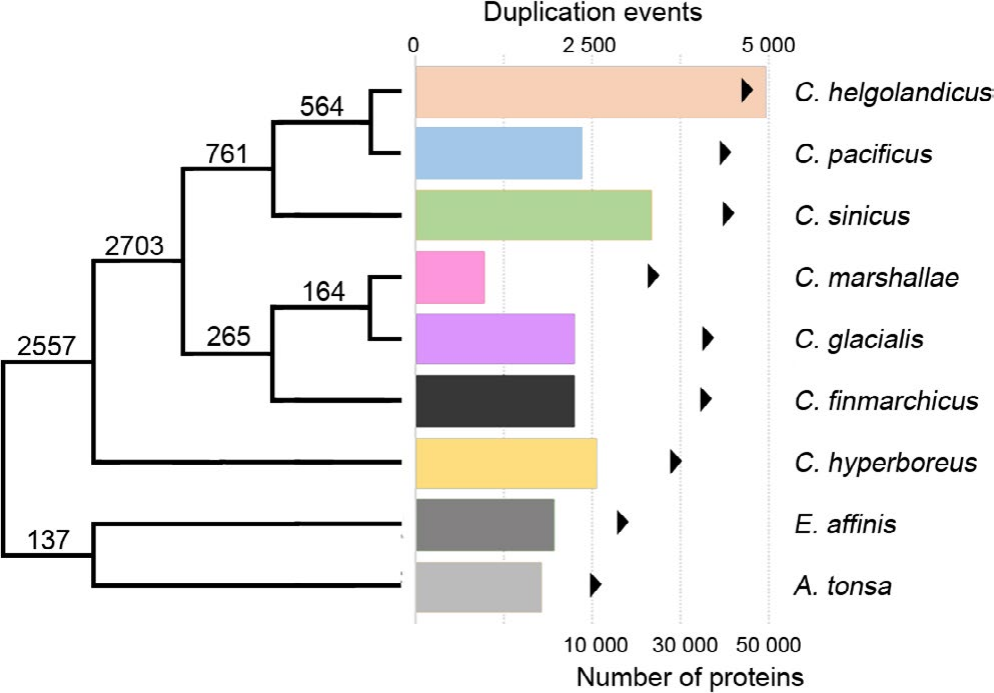
Inferred number of gene duplication events along *Calanus* species tree. Numbers on each branch are duplication events of each respective branch that are retained in all descendant species. Bar plots represent the number of gene duplication events for each species. Black arrows indicate number of proteins per species used for the inference

On average, Orthofinder assigned 32,446 (91%) genes to orthogroups in each *Calanus* species, while 11,610 (84%) genes were assigned to orthogroups in the two outgroup species, meaning that taxon sampling was sufficient. The number of genes in species‐specific orthogroups ranged from 123 in *C*. *marshallae* to 898 in *C*. *helgolandicus* with a mean of 498 genes (1.3%). Estimated duplication events inferred by Orthofinder showed that the largest number of duplication events happened prior to the emergence of the genus *Calanus* and after the split of *C. hyperboreus* (Figure 3). The *C*. *helgolandicus* group had the most duplication events with the most duplication (4970) inferred for *C*. *helgolandicus* itself. The lowest number of duplications was observed in *C*. *marshallae* (988). We also observed that species with the highest number of duplication events are also the species with the highest number of peptides and sequencing output (Tables 2 and S2). Notably, a strong positive correlation is detected between the number of final peptides in each species and the number of estimated duplications (Pearson’s *r* = .82, *p* value = .023).

**FIGURE 4:**
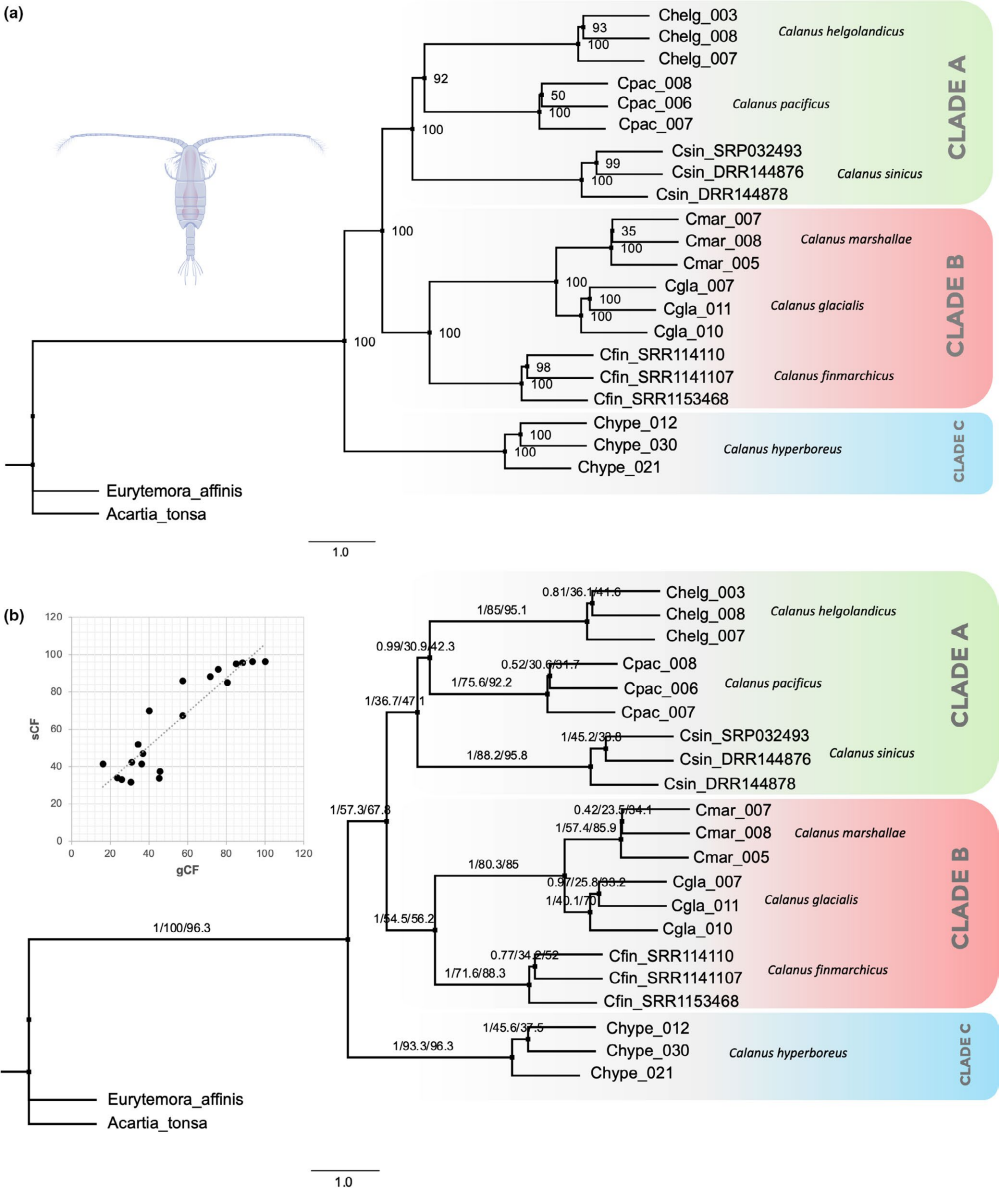
(a) Maximum‐likelihood (ML) phylogenetic tree of seven *Calanus* species and two outgroup taxa *Acartia tonsa* and *Eurytemora affinis* based on 191 single‐copy orthologs derived from transcriptomes. ML tree showing bootstrap support values that are at the maximum on the majority of nodes (ML bootstrap = 100) except *C*. *helgolandicus* & *C*. *pacificus* (bootstrap = 92%). (b) Corresponding ML tree for the *Calanus* spp. dataset including two outgroup taxa. Numbers on each branch represent maximum‐likelihood support value, gCF, and sCF. The inset shows the scatterplot of gCF and sCF values

Orthofinder identified 191 single‐copy protein coding orthologous genes across seven species of *Calanus* and the two outgroup taxa *A*. *tonsa* and *E*. *affinis*. The generated ML trees for each gene ortholog showed that the seven species were clustered into three well‐supported clades based on bootstrap support values (*C*. *sinicus* (*C*. *helgolandicus* + *C. pacificus*); (*C*. *finmarchicus* (*C*. *glacialis* + *C. marshallae*); (*C. hyperboreus*). Moreover, *C*. *sinicus* shared a recent common ancestor with the monophyletic species *C*. *helgolandicus* and *C*. *pacificus*. *C*. *finmarchicus* shared a recent common ancestor with *C*. *glacialis* and *C*. *marshallae*, and there was a consistent split between *C. hyperboreus* and the two other monophyletic groups (*C*. *helgolandicus* and *C*. *finmarchicus* groups, Figure 4a).

Gene and site concordance analyses revealed branches that show a concordant and disconcordant gene and site variations within our reference ML tree (Figure 4b). The split between *C. hyperboreus* group and the two other groups (*C*. *finmarchicus* and *C*. *helgolandicus* group) showed a 100% maximum‐likelihood value with a gCF of 100% and sCF of 96.3%. This means that all 191 protein‐coding genes support this grouping, with most of the amino acid sites informative for this ML branch. Meanwhile, a gCF value of 57.3% or 109 out of 191 single‐copy orthologous genes support the split between *C*. *finmarchicus* and *C*. *helgolandicus* groups with most of the sites (67.8%) informative of the branch topology. 36.7% or ~70 single‐copy orthologs support the separation of *C*. *sinicus* from *C*. *helgolandicus* and *C*. *pacificus*. A third of all single‐copy protein‐coding genes (30.9% gCF) indicate that *C*. *helgolandicus* and *C*. *pacificus* are sister species and about 54.5% of the genes supported the split of *C*. *finmarchicus* with *C*. *glacialis* and *C*. *marshallae* with 56.2% of these protein‐coding genes containing informative sites. Furthermore, the scatter plot of gCF and sCF values for all branches shows that the majority of single‐copy orthologs used to reconstruct the species tree contained informative gene and site information.

## 4 DISCUSSION

### 4.1 15 new de novo transcriptome assemblies for *Calanus* spp.

With the aim of contributing to and improving the existing transcriptomic resources available for the genus *Calanus*, we analyzed the transcriptomes of five species of *Calanus* together with two outgroups and two *Calanus* species mined from NCBI database. Overall, we were able to contribute to the existing online high‐throughput sequencing database with 15 new transcriptomes, including two species never sequenced before (*C. hyperboreus* and *C. marshallae*). The quality of our transcriptome assemblies was similar, if not slightly higher, than previously assembled de novo transcriptomes for *Calanus* spp. according to several metrics. For instance, the mean percentage of alignment among the individuals generated from the present study was 96.69%, while the mean percentage alignment among the six de novo transcriptome assemblies that we downloaded from NCBI database is 93.08%. Our results satisfy the currently accepted criteria that a good Trinity transcriptome assembly should have a high percentage alignment or that most of the reads should map back to the assembly. This is also true with the Nx50 value where the mean Nx50 from our study is 1118.8, while the mean Nx50 among database samples is 1007.83. Furthermore, we also computed for the ExN50 (i.e., N50 based only on the topmost highly expressed genes), considered to be one of the most appropriate metrics to assess transcriptome data quality (Haas et al., 2013). Our ExN50 ranged between 2257 and 2854 with a mean value of 2438 (Figure S4). Lastly, to complement the technical metrics of N50 statistics, we used BUSCO to indirectly assess our assembly completeness using a specific set of near‐universal single‐copy orthologs based on the BUSCO Arthropoda database. Our results show a relatively complete assembly among the 15 individuals with a mean BUSCO completeness value of 90.55%. Both the summary statistics and BUSCO measurements showed similar or higher values compared to previously published studies related to de novo transcriptome assemblies in *Calanus* spp. and other copepods (Berger et al., 2021; Lenz et al., 2014; Tarrant et al., 2014, 2019; Yang et al., 2014). Although the transcriptomes presented here are of high quality, they remain partially incomplete because they only represent one or two specific developmental stages of a species in a snapshot of natural conditions and are likely lacking stage‐specific, condition‐ or stress‐specific transcripts. More sequencing efforts are needed to further improve transcriptomic resources for the genus *Calanus*.

The recent Ocean ZOOP initiative (Ocean Zooplankton Open ‘Omics Project) has called for multispecies high‐quality de novo transcriptomes for zooplankton species spanning diverse taxa from across the world's oceans, to generate a new framework for evolutionary, ecological, and physiological studies (Lenz et al., 2021). Our results on multiple *Calanus* species will thus contribute to such most needed project. In addition, here we demonstrated how the use of whole transcriptome data can help resolve evolutionary relationships among closely related zooplankton species.

### 4.2 Conserved protein functions across species

Functional assignment based on COG and GO term annotations showed a conserved pattern of protein functions across different *Calanus* species. In particular, we noticed that relatively few protein families encode for functions relating to nuclear structure compared to other eukaryotic species (i.e., *Arabidopsis thaliana*, *Caenorhabditis elegans*, *Drosophila melanogaster*, *Homo sapiens*, and *Saccharomyces cerivisae* Tatusov et al., 2003). This COG pattern is also distinct from other distantly related marine organisms such as the fish *Coilia nasus* (Du et al., 2014); the diatom *Skeletonema costatum* (Zhang et al., 2016); and the crab *Eriochier sinensis* (Li et al., 2013). The results of our COG annotation indicate a lack of protein families in functional categories N (Cell motility) and Y (Nuclear structure). However, this may be linked to their small numbers in the database compared to protein families in other COG categories, and slightly lower efficiency of automated annotation compared to manual, but not necessarily to the absence of these proteins in *Calanus* transcriptomes. We also found that 24.3% of the protein families among seven *Calanus* species do not have a known function and that no proteins were functionally assigned to the general function predictions (R) category. Our results are almost similar with the COG annotation performed by Yang et al., 2014 for *C*. *sinicus* except that we were able to assign more protein families with functions related to extracellular structures (393–677 protein queries vs. <100, for *C. sinicus*, Yang et al., 2014). In general, the total number of protein families assigned to known functions (based on COG annotation) was much lower (~6383 out of 43,417 protein queries assigned) in the study of Yang et al. (2014), due to the limited number of reference genomes present in the COG database back in 2014. GO annotation also showed that sequenced and assembled transcriptomes of *Calanus* species have patterns of annotated transcripts similar to previously sequenced and here re‐assembled *C*. *finmarchicus* (Tarrant et al., 2014), *C*. *sinicus* (Yang et al., 2014), and other recently investigated copepods, for example, *Labidocera madurae* (Roncalli et al., 2017) and *Rhincalanus gigas* (Lauritano et al., 2020).

### 4.3 Orthologs and duplication events

According to Orthofinder, most protein‐coding genes are conserved among the seven *Calanus* species with only 1.3% of the genes being species‐specific. This percentage of species‐specific genes is a bit higher than what was reported for five species of bats in the recent study of (Moreno‐Santillán et al., 2019) and for five species of lizards in (Maldonado et al., 2020). The number of duplication events are expected to be high for *Calanus* copepods due to their large genomes (McLaren et al., 1988) and extended gene families (e.g., Lenz et al., 2014). Surprisingly, the largest number of duplication events was inferred in *C*. *helgolandicus* and other species of the *C*. *helgolandicus* group (*C*. *pacificus* and *C. sinicus*). It was only intermediate in *C. hyperboreus* and *C*. *glacialis*, despite them having the largest genome size estimations (McLaren et al., 1988). The lowest number of duplication events was found in *C*. *marshallae*, which has a genome size estimate comparable to that of *C*. *helgolandicus*. The strong positive correlation between duplication events and the number of predicted peptides used as input suggests that these results could be biased by sequencing effort and completeness of species transcriptomes. However, the positive correlation between the number of peptides and duplication events does not shed light on this question and only sequenced genomes of *Calanus* species will unequivocally resolve this issue.

### 4.4 Fully resolved phylogenetic relationships for seven *Calanus* species

In the present study, we were able to reconstruct the phylogenetic relationships of seven species of *Calanus* based on 191 single‐copy protein‐coding orthologs from RNA‐seq data. The results of our maximum‐likelihood tree indicated that these seven species cluster into three well‐supported clades. Our phylogenetic analysis also showed a fully resolved *C*. *helgolandicus* group (*C*. *sinicus* as a sister clade to the sister species *C*. *helgolandicus* and *C*. *pacificus*, clade A) in comparison with the earlier study of Bucklin et al. (1995) using 16S rRNA, wherein several species of the *C*. *helgolandicus* group (*C. helgolandicus*, *C. pacificus*, and *C*. *sinicus*) did not group together well, largely because of the ambiguous position of *C*. *pacificus*. The relationships within the *C*. *helgolandicus* group relied on a single molecular marker and were only loosely supported by the bootstraps (Bucklin et al., 1995). Moreover, our phylogenetic results slightly disagree with the phylogenetic tree from Kozol et al. (2012) based on 658 bp region of the 28S rRNA gene, where the authors found that *C*. *helgolandicus* is a sister species to *C*. *pacificus* and *C*. *sinicus*, while our ML tree suggests that *C*. *helgolandicus* and *C*. *pacificus* are more related than *C*. *pacificus* and *C*. *sinicus* are. Our tree topology was supported by high bootstrap values (bootstrap = 100, *C*. *sinicus* (*C*. *pacificus* + *C. helgolandicus*); and bootstrap = 92, *C*. *pacificus* + *C. helgolandicus*), with more individuals per species and higher number of genes (~60 to 70 single‐copy orthologs supporting these branches) compared to earlier studies. Meanwhile, species relationships in both Clade B and Clade C are consistent with all the previously proposed *Calanus* phylogenies (Bucklin et al., 1995; Frost, 1974; Kozol et al., 2012).

The topology of our ML tree was more concordant with the topology found by Frost (1974) and by Fleminger (reported in Bucklin et al., 1995) based on the examination of several morphological characters. Our study demonstrates the power that can be obtained with large molecular datasets to fully resolve phylogenies, in comparison with using only a single genetic marker. The concordance between morphology‐based and well supported/confirmed molecular phylogenies is particularly interesting in the case of *Calanus* spp., as the strong morphological similarity between species within the genus has challenged the work of taxonomists for decades, especially for species within Clade B. Recently, a thorough assessment of morphological characters considered to be diagnostic for species discrimination between *C*. *finmarchicus* and *C*. *glacialis* was made using genetic tools and revealed that most if not all morphological characters were unreliable, depending on geographical location (Choquet et al., 2018). To reconstruct the *Calanus* phylogeny, Frost (1974) examined several morphological characters including: the fifth pair of swimming legs, the relative size of accessory photoreceptor, the length of caudal ramus, the size of genital pore, and the shape of the ventral surface of the genital segment. Some of these taxonomic characters have been re‐investigated recently together with some genetic information (mostly between *C*. *glacialis* and *C*. *finmarchicus* species). No evident species‐specific patterns were observed, and results are shown to be species independent (e.g., 5th pair of swimming legs Choquet et al., 2018; secondary sexual structures—K. Kosobokova, *personal communication*) or significantly variable depending on geography. However, the combined analysis of multiple morphological traits, as performed in the study of Frost (1974) from individuals sampled in regions where species‐specific morphological differences may be more distinct (*see* Choquet et al., 2018) could have contributed to a more similar phylogenetic tree between the phylogeny based on morphology and the phylogeny based on new transcriptome datasets (this study).

The taxonomic status of *C*. *marshallae* has been in question due to the extreme similarity of mtCOI and mt16S sequences with that of *C*. *glacialis* reported in GenBank. Minimal differences in barcode sequences have raised doubts among experts about the current taxonomic status of *C*. *marshallae* species. Ashjian et al. (2017) reported a single base pair difference between species in the COI mitochondrial gene across 1500 specimens of *C. glacialis*/*C*. *marshallae* analyzed in their study. Their genetic analyses revealed three genetically differentiated groups (Ashjian et al., 2017): *C*. *marshallae* from Puget Sound (identified by B. Frost), the Arctic *C*. *glacialis* collected at SHEBA ice camp in Canadian basin (Ashjian et al., 2003), and *C*. *glacialis* samples from the Bering Sea. Here, our data suggest a strong genetic differentiation between *C*. *glacialis* (collected from Skjerstadfjord) and *C*. *marshallae* (from Puget Sound c/o B. Frost) and may indicate that they are two distinct species, notably because of the high bootstrap support value and concordance factor observed in the branch separating these taxa (ML bootstrap = 100%; gCF = 80.3%; sCF = 85%), relative to the other species present in our phylogenetic analysis. However, we could not investigate further the taxonomic status of the three genetically differentiated clusters reported by analyses of mtCOI (Ashjian et al., 2017) since we did not include samples of *C*. *glacialis* from the Bering Sea. More individuals identified as *C*. *marshallae* and *C*. *glacialis* from these three localities must be analyzed to further assess the actual taxonomic status of these three genetic clusters. Combining comprehensive morphological examinations together with analyses of large numbers of genome‐wide molecular markers such as single nucleotide polymorphisms (SNPs), following the genome‐reduced representation protocol developed by Choquet et al. (2019), will allow testing for reproductive isolation among these three genetic entities.

Lastly, to get more insights on our ML tree, we quantified genealogical concordance in our phylogenetic dataset. Gene concordance factor (gCF) is defined as the percentage of decisive gene trees supporting a branch, while sCF is the percentage of decisive alignment sites supporting a branch in a reference tree (Minh et al., 2020). These concepts are important for phylogenomics datasets because genes that are from different chromosomes or from distant regions of the genome tend to show different levels of resolution or phylogenetic signals (see Minh et al., 2020; Rota et al., 2021). The results of our concordance estimations fully supported the split between *C. hyperboreus* and the two other *Calanus* groups: *C*. *finmarchicus* and *C*. *helgolandicus* (gCF = 100%; sCF = 96.3%). We also found that 109/191 single‐copy protein‐coding genes supported the separation between *C*. *finmarchicus* and *C*. *helgolandicus* groups. Although we obtained the maximum possible bootstrap support values for each branch of our ML tree, we still observed low gCF and sCF values for branches splitting the three species in the *C*. *helgolandicus* group and in the shallower splits in general. High bootstrap values, but somewhat low concordance factors between lineages are common and have also been observed in several studies using gene orthologs derived from transcriptomes (i.e., in frogs, Chan et al., 2020; in spiders, Kallal et al., 2020; in butterflies Rota et al., 2021; in ants van Elst et al., 2021). Low concordance values do not mean that the phylogenetic tree is unresolved, but rather gives us further insights on how related or congruent the genes are in resolving the species phylogeny (Minh et al., 2020). In addition, the relatively low concordance factors in the shallower branches of our phylogenetic tree may be attributed to conflicting signals among the 191 single‐copy orthologs used to reconstruct our phylogenetic tree (Minh et al., 2020). Unfortunately, this issue of concordance between multiple genomic markers represents an obstacle for the calculation of divergence time within the genus *Calanus* and needs to be resolved by either clustering genes with similar phylogenetic signals or by using a different set of genomic markers from transcriptome datasets such as SNPs. Nevertheless, our study provides the most recent, well‐resolved and multigenic phylogenetic analysis for copepod species of the genus *Calanus* in the northern seas.

## 5 CONCLUSION

The use of RNA‐sequencing enabled us to contribute and improve the existing transcriptome database for the genus *Calanus* as well as build a baseline information for future comparative transcriptomics in evolutionary and eco‐physiological contexts. Our study is the first attempt to utilize phylotranscriptomics to resolve species relationships among the *Calanus* species living in the North Atlantic, North Pacific, and Arctic Oceans, and in copepods in general. This resulted in the reconstruction of a much‐improved phylogenetic tree and clarification of certain ambiguities within the *Calanus* genus. Moreover, the phylogenetic tree inferred in this study showed the potential of using concordance factor to look at underlying variations in phylogenomics data beyond the limitations of bootstrapping method. As phylotranscriptomic analyses are getting more accessible and popular, more robust and streamlined, improvements in the ease of analyses and development of a consensus in interpretation of data shall be expected in the near future.

## CONFLICT OF INTEREST

The authors declare they have no conflict of interest.

## AUTHOR CONTRIBUTION

**Apollo Marco Dalonos Lizano:** Conceptualization (equal); Data curation (equal); Formal analysis (equal); Investigation (equal); Methodology (equal); Software (equal); Validation (equal); Visualization (equal); Writing – original draft (equal); Writing – review & editing (equal). **Irina Smolina:** Conceptualization (equal); Formal analysis (equal); Methodology (equal); Software (equal); Visualization (equal); Writing – review & editing (equal). **Marvin Choquet:** Conceptualization (equal); Project administration (equal); Supervision (equal); Writing – review & editing (equal). **Martina Kopp:** Methodology (equal); Project administration (equal); Resources (equal). **Galice Hoarau:** Conceptualization (equal); Funding acquisition (equal); Project administration (equal); Supervision (equal); Writing – original draft (equal); Writing – review & editing (equal).

## Supporting information

Fig S1

Fig S2

Fig S3

Fig S4

Appendix S1

## Data Availability

RNA‐seq reads used for assembling of 15 transcriptomes are available at NCBI SRA database under BioProject PRJNA744376 and 15 individual de novo transcriptomes generated on this project has been deposited at DDBJ/EMBL/GenBank TSA database and DRYAD server (https://doi.org/10.5061/dryad.n8pk0p2ww).
