# Supplementary figures and images for: Insights into the species evolution of Calanus copepods in the northern seas revealed by de novo transcriptome sequencing
Source: Ecol Evol. 2022 Feb 22;12(2):e8606. doi: 10.1002/ece3.8606 (PMC8861592; doi:10.1002/ece3.8606)

## Species

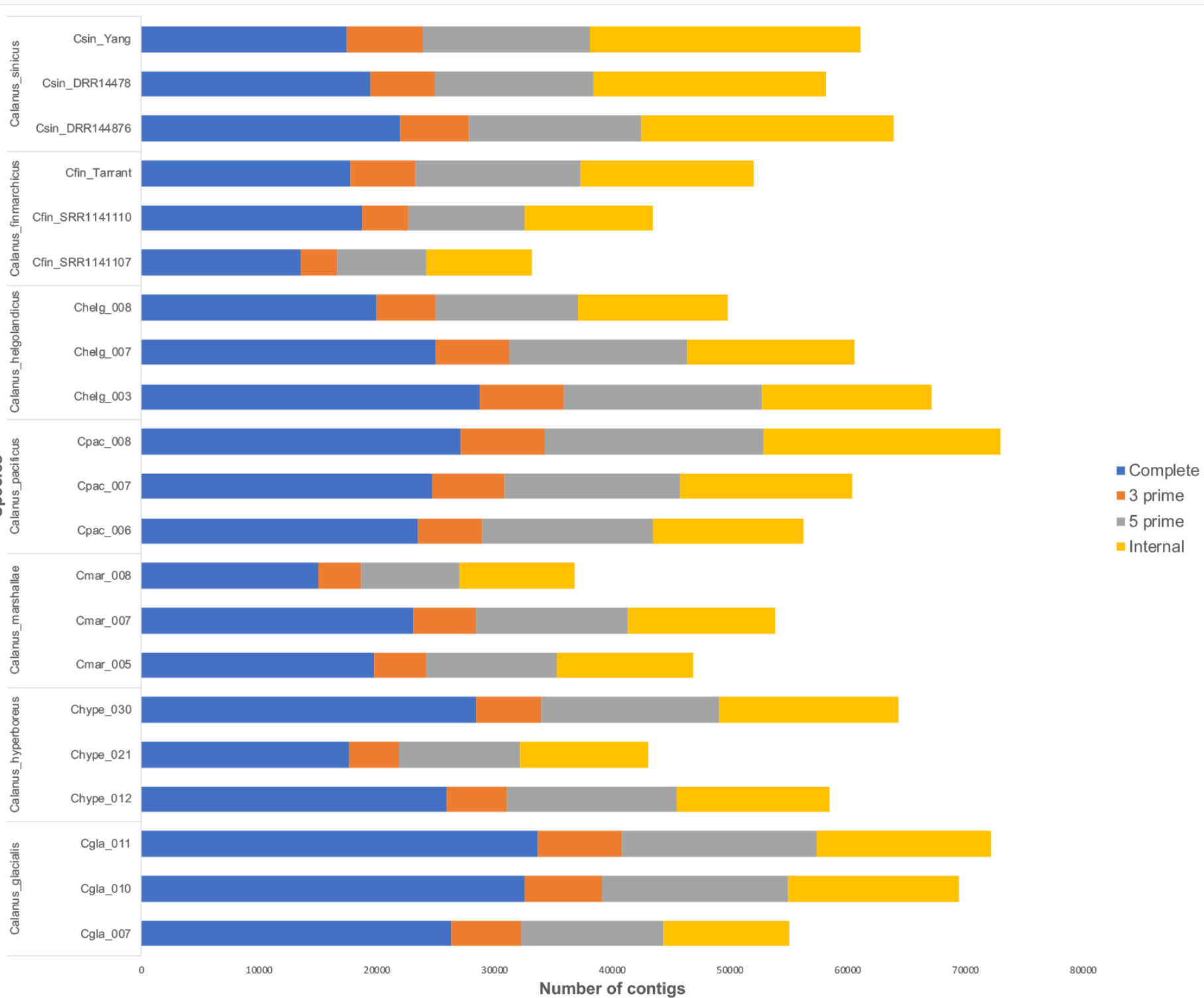

Supplement: Supplementary file 1 — Fig S1 [file ECE3-12-e8606-s002.pdf]

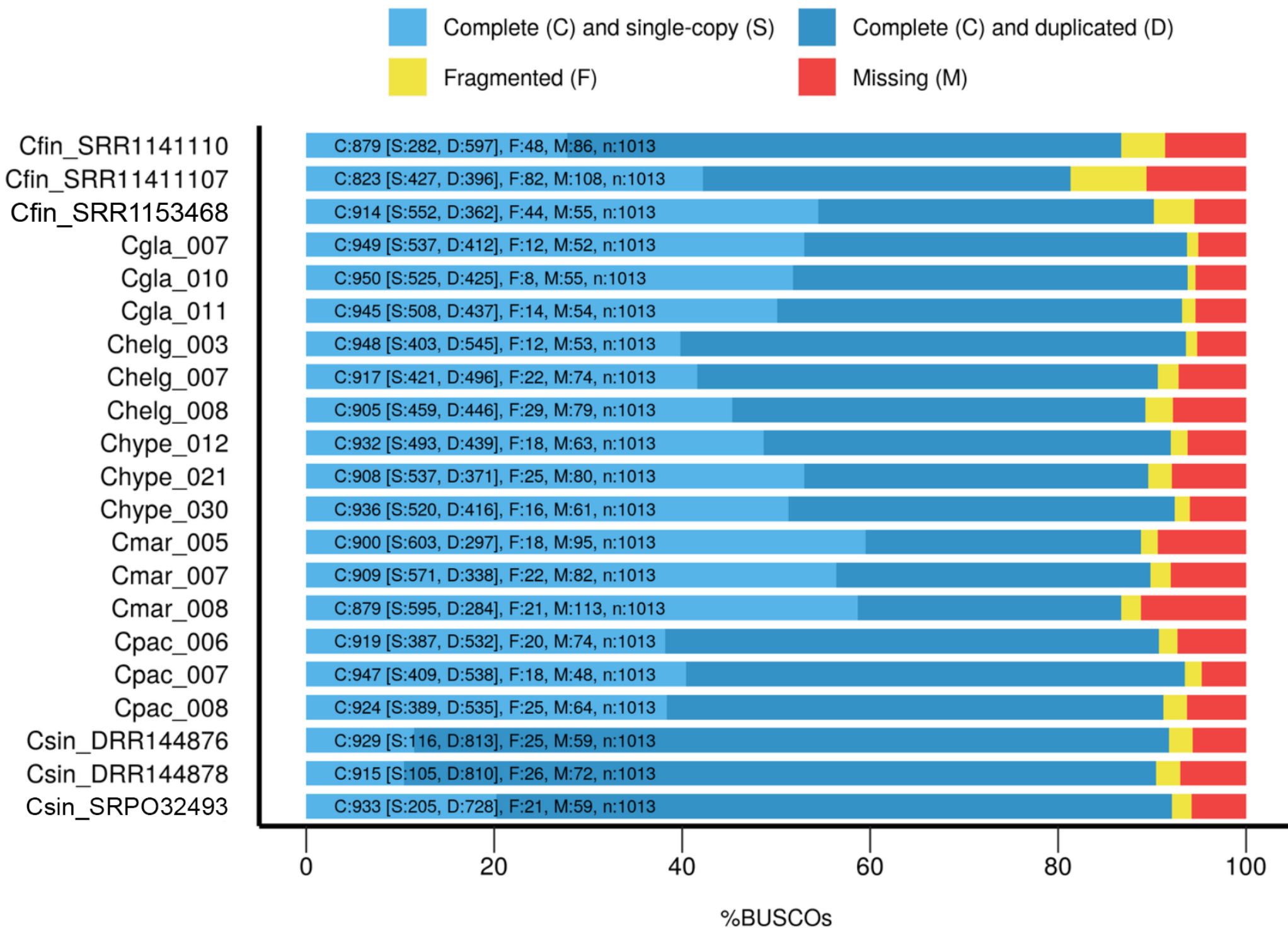

Supplement: Supplementary file 2 — Fig S2 [file ECE3-12-e8606-s003.pdf]

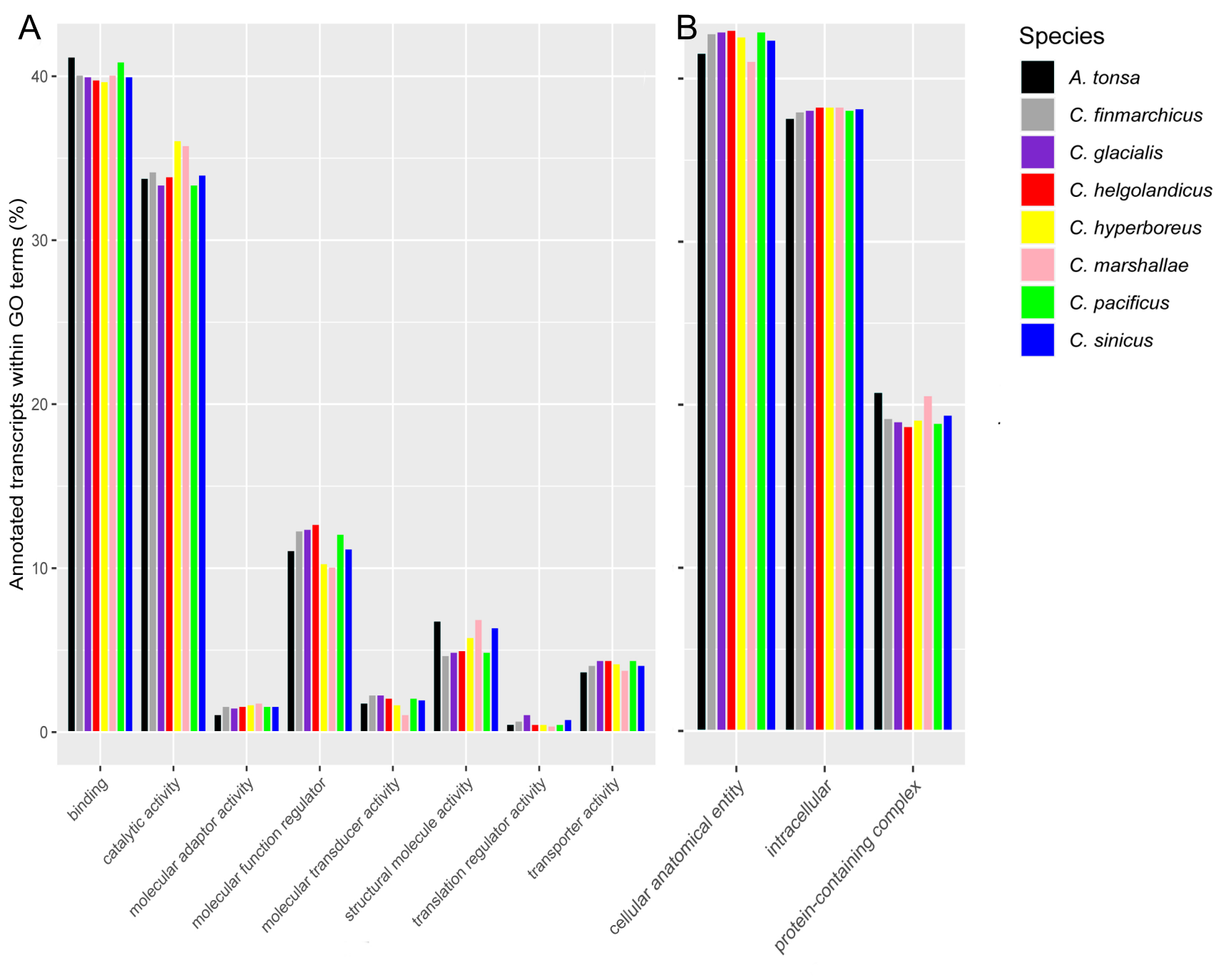

Supplement: Supplementary file 3 — Fig S3 [file ECE3-12-e8606-s004.pdf]

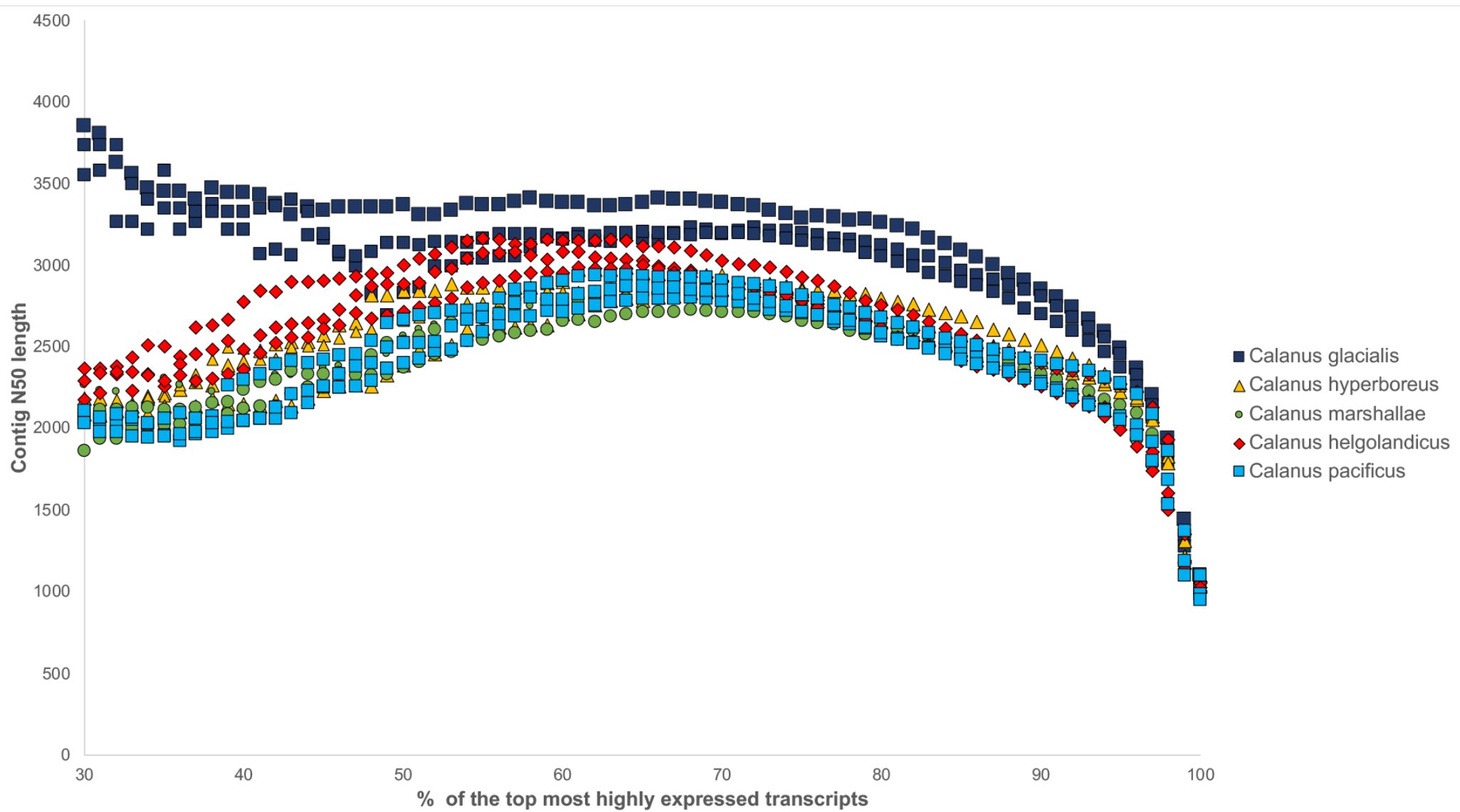

Supplement: Supplementary file 4 — Fig S4 [file ECE3-12-e8606-s005.pdf]
