# Supplementary material for: Insights into the species evolution of Calanus copepods in the northern seas revealed by de novo transcriptome sequencing
Source: Ecol Evol. 2022 Feb 22;12(2):e8606. doi: 10.1002/ece3.8606 (PMC8861592; doi:10.1002/ece3.8606)
Supplement: Supplementary file 5 — Appendix S1 [file ECE3-12-e8606-s001.pdf]

Appendix

Figures

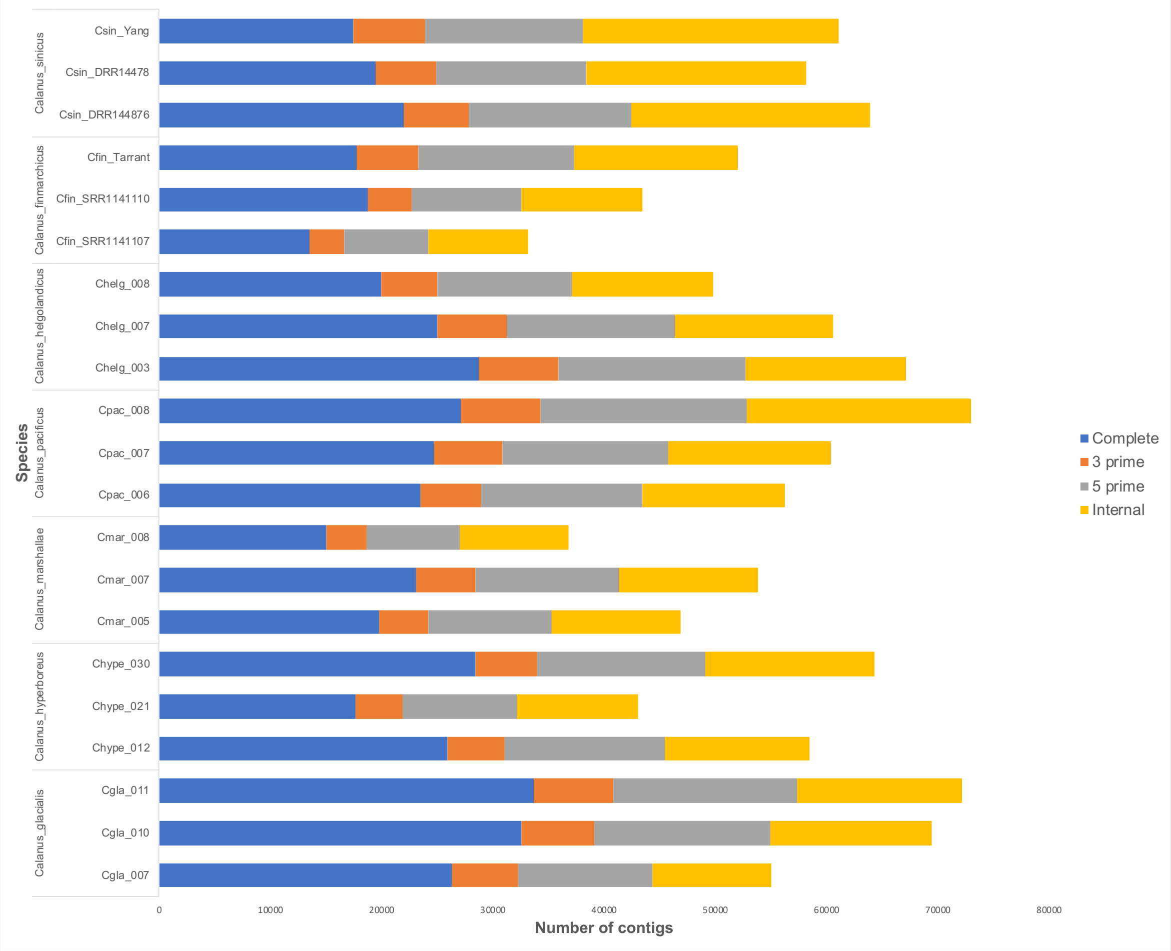

Figure S1. Number of transcripts that are at least 100 bp long and containing open reading frames (ORFs) identified using Transdecoder pipeline. “Complete” means that both start and stop codon are present, “3 prime” refers to partial ORF lacking a start codon, “5 prime” represents contigs without stop codon, and internal ORF lack both the stop and start codon.

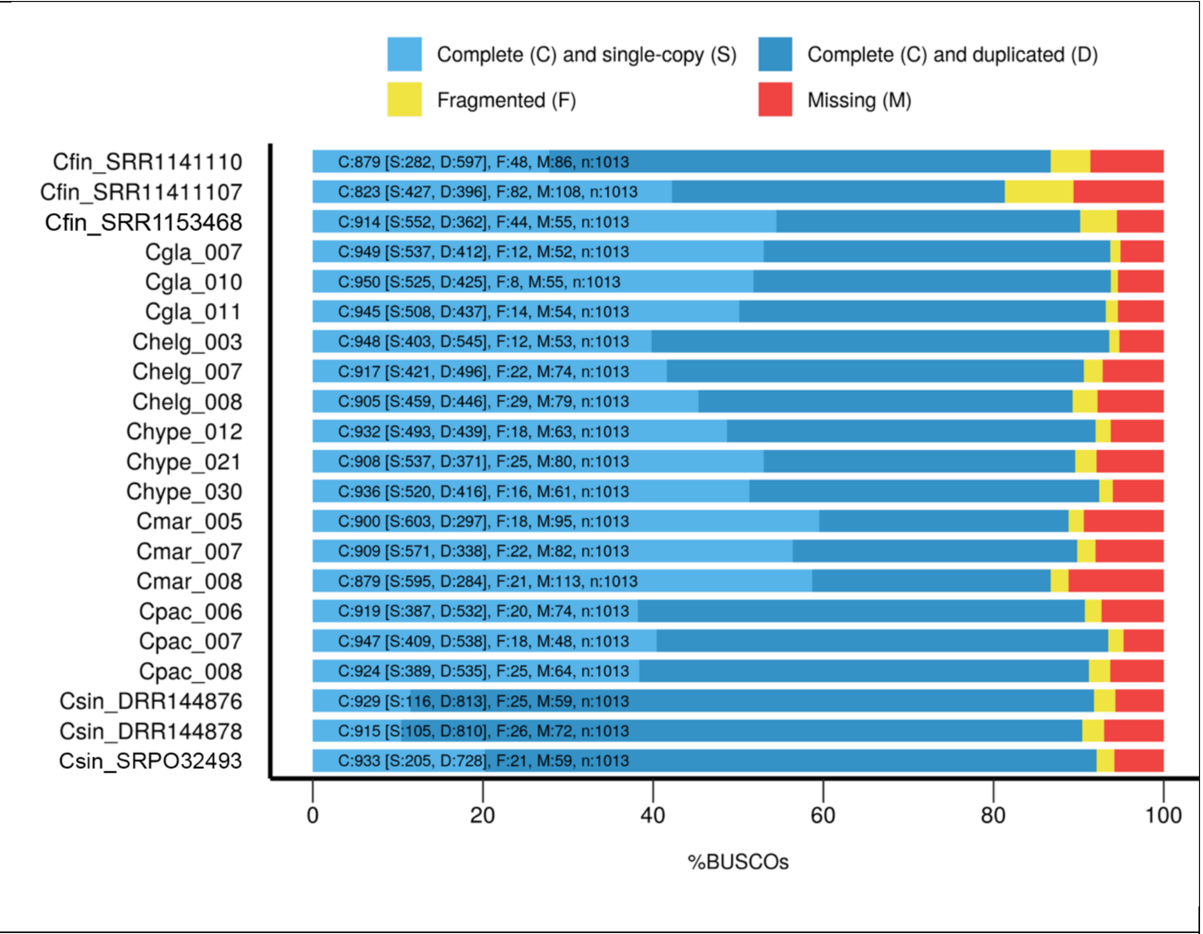

Figure S2. Percentage of complete single-copy, duplicated, fragmented, and missing orthologs inferred from BUSCO arthropoda database. Cfin = *Calanus finmarchicus,* Cgla = *C. glacialis, Chelg* = *C. helgolandicus, Chype* = *C. hyperboreus, Cmar* = *C. marshallae, Cpac*= *C. pacificus, Csin* = *C. sinicus.*

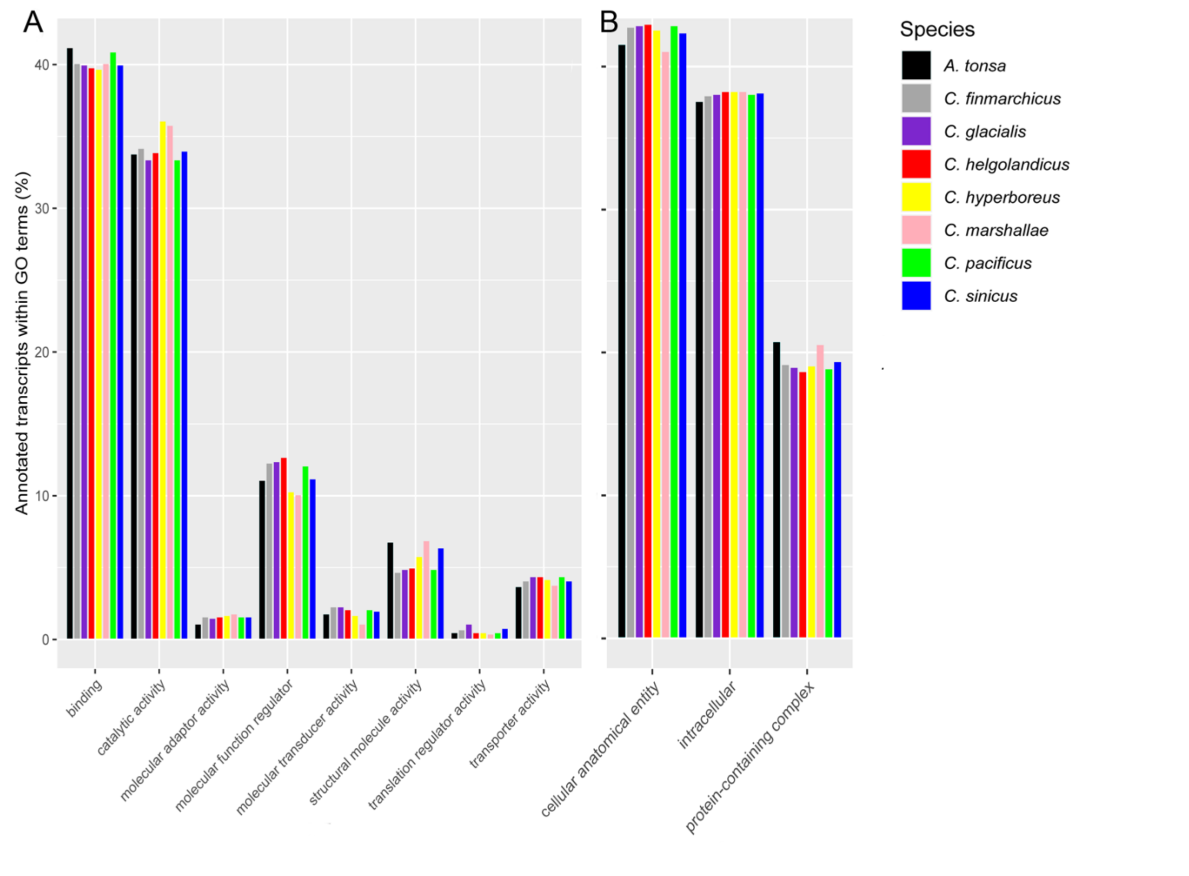

Figure S3. Gene ontology (GO) annotation representing A. molecular function (MF) and B. cellular component (CC).

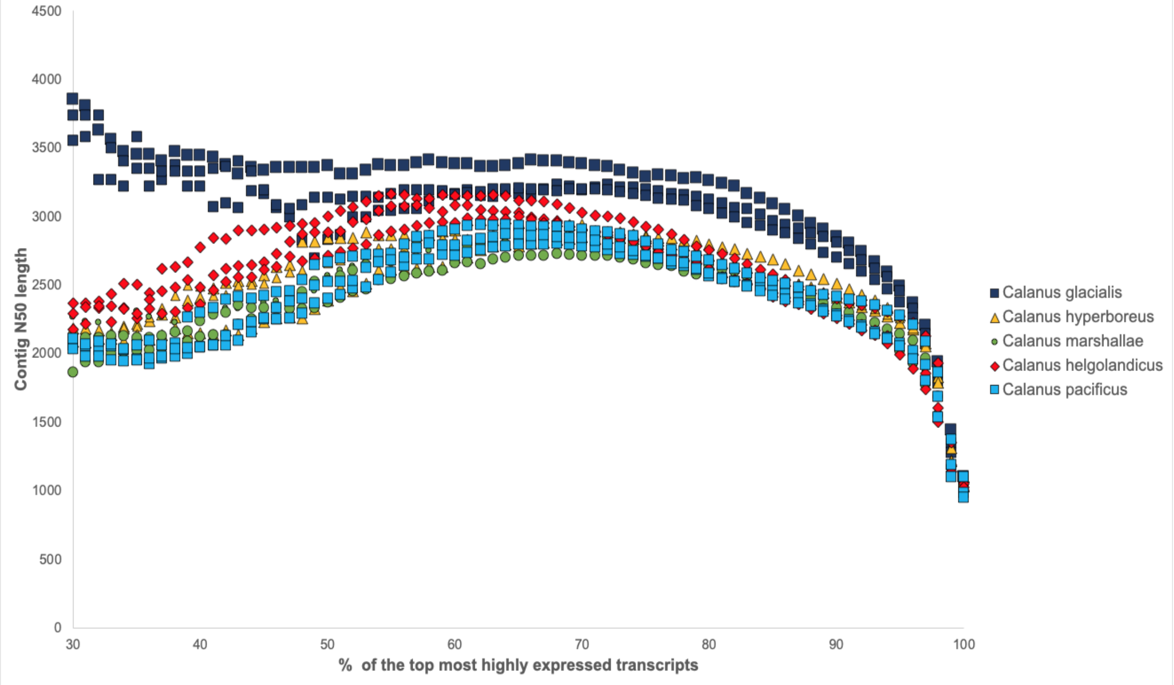

Figure S4. ExN50 plot of ExN50 length with regards to percentage of the top-most highly expressed transcripts for *Calanus* species generated via transcriptome assembly.

Tables

Table S1. Summary of all transcriptome datasets available at NCBI SRA database as of December 2020

Number	Species	Data type	Scope	Resources	Accession	Title	Samples	Registration date	Publication citation	Sequencing
1	*Calanus sinicus*	Transcriptome or Gene expression	Monoisolate	The University of Tokyo, Atmosphere and Ocean Research Institute	PRJDB7229	Identification of molecular markers associated with starvation or diapause in copepods	3 Whole samples from satiated *Calanus sinicus;* 3 whole samples from starved *Calanus sinicus*	19-Jul-20	Ohnishi et al. 2019	Illumina HiSeq 4000; 100bp PE
2	*Calanus helgolandicus*	Raw sequence reads	Multispecies	Stazione Zoologica Anton Dohrn di Napoli	PRJNA640515	De Novo Transcriptome Assembly and Gene Expression Profiling of the Copepod *Calanus helgolandicus*	6 samples of adult female *Calanus helgolandicus*	19-Jun-20	Asai et al. 2020	Illumina HiSeq 2000; 50bp PE
3	*Calanus finmarchicus*	Transcriptome or Gene expression	Multiisolate	Carl von Ossietzky University	PRJNA628886	*Calanus finmarchicus* transcriptome	42 samples containing	28-Apr-20	Payton et al. 2020	NovaSeq S4 lane

				of Oldenburg		during the Arctic summer solstice	15 pooled CV *Calanus finmarchicus* adults per sample			, PE 2x150bp
4	*Calanus finmarchicus*	Raw sequence reads	Multiisolate	NTNU	PRJNA420690	*Calanus* development and responses to WSF of petrogenic oil	4 Adult female and 8 CV adult *Calanus finmarchicus*	01-Dec-17	Skotene et al. 2019a; Skotene et al. 2019b	Illumina HiSeq 500; 75bp PE
5	*Calanus finmarchicus*	Raw sequence reads	Multiisolate	Woods Hole Oceanographic Institution	PRJNA358233	NA	RNA-seq of *Calanus* nauplii exposed to different with varied oil exposure	20-Dec-16	Hansen et al. 2017	Illumina HiSeq 2000
6	*Calanus finmarchicus*	Transcriptome or Gene expression	Environment	University of Hawaii at Manoa	PRJNA356331	*Calanus finmarchicus* late nauplii (NV-NVI) transcriptome of Alexandrium fundyense response	3 *Calanus finmarchicus* late nauplii feeding on *Rhodomonas* sp. And 3 C. *finmarchic*	05-Dec-16	Roncalli et al. 2017	Illumina HiSeq 2000; PE

							us late nauplii feeding on *Alexandrium fundyense*			
7	*Calanus glacialis*	Transcriptome or Gene expression	Multiisolate	Norwegian Polar Insitute	PRJNA352656	NA	23 *Calanus glacialis* samples containing 10 pooled nauplii	07-Nov-16	NA	Illumina HiSeq 2500; SE
8	*Calanus pacificus*	Transcriptome or Gene expression	Multiisolate	Oregon State University	PRJNA335617	*Calanus pacificus* Transcriptome or Gene expression	13 pooled adult females *Calanus pacificus*	27-Jul-16	NA	Illumina MiSeq; 150 PE
9	*Calanus finmarchicus*	Transcriptome or Gene expression	Monoisolate	University of Hawaii at Manoa	PRJNA328961	*Calanus finmarchicus* 2012 Developmental Stages Raw sequence reads	13 Different stages of *Calanus finmarchicus*	13-Jul-16	Tarrant et al. 2014	Illumina HiSeq 2000; PE
10	*Calanus finmarchicus*	Transcriptome or Gene expression	Multispecies	Harvard University	PRJNA328606	A Phylogenomic Solution to the Origin of Insects by	1 individual - RNAseq of *Calanus finmarchic*	12-Jul-16	Schwentner et al. 2017	Illumina HiSeq 2500; PE

						Resolving Crustacean-Hexapod Relationships	*us* using whole animal			
11	*Calanus glacialis*	Transcriptome or Gene expression	Monoisolate	Centre of Marine Sciences, CCMAR	PRJNA274584	A transcriptome resource for the copepod *Calanus glacialis* across a range of culture temperatures	6 samples of *Calanus glacialis* with different culture temperatures	04-Feb-15	Ramos et al. 2015	454 GS FLX; SE; random PCR
12	*Calanus glacialis*	Transcriptome or Gene expression	Multiisolate	Nord University	PRJNA237014	Contrasting transcriptome response to thermal stress in two key zooplankton species, Calanus finmarchicus and C. glacialis	6 samples of *Calanus finmarchicus* exposed at different temperatures	30-Jan-14	Smolina et al. 2015	Ion-Torrent PGM; SE
13	*Calanus finmarchicus*	Transcriptome or Gene expression	Multiisolate	Nord University	PRJNA236983	Contrasting transcriptome response to thermal stress in two key zooplankton species,	7 samples of Calanus glacialis exposed at different	30-Jan-14	Smolina et al. 2015	Ion-Torrent PGM; SE

						Calanus finmarchicus and *C. glacialis*	temperatures			
14	*Calanus finmarchicus*	Transcriptome or Gene expression	Multiisolate	Pacific Biosciences Research Center, University of Hawaii at Manoa	PRJNA236528	Glutathione S-Transferase (GST) Gene Diversity in the Crustacean *Calanus finmarchicus*--Contributors to Cellular Detoxification	48 samples of *Calanus finmarchicus* from the Arctic Ocean	27-Jan-14	Roncalli et al. 2015	Illumina NovaSeq 6000; PE
15	*Calanus sinicus*	Transcriptome or Gene expression	Monoisolate	National Marine Environment Monitoring Center	PRJNA225989	Development of molecular resources of the copepod *Calanus sinicus* for its potential ecological applications via a comprehensive transcriptome study	1 individual *Calanus sinicus*	01-Nov-13	Yang et al. 2014	Illumina HiSeq 2000; PE

16	*Calanus sinicus*	Transcriptome or Gene expression	Multiisolate	Institute of oceanology, Chinese academy of sciences	PRJNA185333	Transcriptome Sequencing and De Novo Analysis for Copepod (*Calanus sinicus)* using 454 GS FLX	1 individual of *Calanus sinicus*	05-Jan-13	Ning et al. 2013	454 GS FLX; SE; RT-PCR
17	*Calanus finmarchicus*	Transcriptome or Gene expression	Multiisolate	Woods Hole Oceanographic Institution	PRJNA231164	Transcriptional profiling of reproductive development, of the marine copepod *Calanus finmarchicus*	17 samples of C5 copepodites stage of *Calanus finmarchicus*	11-Dec-13	Lenz et al. 2014	Illumina HiSeq 2000; PE

Table S2. RNA sequencing results for the 21 *Calanus* transcriptomes generated in this study.

Individual name	Species	Output size after demultiplexing (gb)	Total no. of raw reads	PE Read length (bp)	C-value (pg) Based on http://genomesize.com/
Chype_012	*Calanus hyperboreus*	26	75858304	151	
Chype_021		11,4	33410124	151	5,51-12,46
Chype_030		19,6	57313368	151	
Cgla_007	*Calanus glacialis*	18,4	54051262	151	
Cgla_010		18	52916092	151	5,22-12,10
Cgla_011		19,6	57514090	151	
Chelg_003	*Calanus heloglandicus*	36	102254447	151	
Chelg_007		13	37891052	151	10,17
Chelg_008		11,4	20820146	151	
Cmar_005	*Calanus marshallae*	8	23456472	151	
Cmar_007		13	38111352	151	10,7 (McLaren et al. 1988)
Cmar_008		3,8	10843622	151	
Cpac_006	*Calanus pacificus*	17	49899236	151	
Cpac_007		22	59434172	151	6,81
Cpac_008		13,8	40561078	151	
		**251**			
Undetermined reads		**24**	163462484		

Table S3. Missing orthologs identified by BUSCO among 15 de novo transcriptomes of 7 *Calanus* species. Proteins with * lists the proteins with query length of <50% to one of translated *Calanus* transcriptomes.

BUSCO ID	Product	Protein accession number
117586at6656	SET and MYND domain-containing protein 5 [Plutella xylostella]	XP_011562133.2
120907at6656	ER membrane protein complex subunit 10 [Plutella xylostella]	XP_011568289.2
*128030at6656	-ketodihydrosphingosine reductase [Plutella xylostella]	XP_037969682.1
132854at6656	glutamate--cysteine ligase regulatory subunit [Vanessa tameamea]	XP_026499379.1
137334at6656	28S ribosomal protein S14, mitochondrial [Copidosoma floridanum]	XP_014215005.1
150479at6656	probable 28S ribosomal protein S25, mitochondrial [Plutella xylostella]	XP_011554200.2

*59393at6656	serine palmitoyltransferase 1 [Plutella xylostella]	XP_037971886.1
99998at6656	protein prenyltransferase alpha subunit repeat-containing protein 1 [Plutella xylostella]	XP_011548970.2

Table S4. NCBI GenBank accession numbers of raw sequences (SRA) and *de novo* transcriptome assemblies (TSA) generated in this study.

**Sample**	**Species**	**Bioproject no.**	**Biosample no.**	**Sequence Read Archive (SRA) accession no.**	**Transcriptome Shotgun Assembly (TSA) project accession no.**
Cgla_007			SAMN24106072	SRR17240412	
Cgla_010	*Calanus glacialis*		SAMN24106073	SRR17240411	GJQS00000000
Cgla_011			SAMN24106074	SRR17240410	
Chelg_003			SAMN24113554	SRR17245871	
Chelg_007	*Calanus helgolandicus*	PRJNA744376	SAMN24113555	SRR17245870	GJQW00000000
Chelg_008			SAMN24113556	SRR17245869	
Cmar_005	*Calanus marshallae*		SAMN24142451	SRR17248906	GJQX00000000
Cmar_007			SAMN24142452	SRR17248905	

Cmar_008		SAMN24142453	SRR17248904	
Cpac_006		SAMN24144303	SRR17249509	
Cpac_007	*Calanus pacificus*	SAMN24144304	SRR17249508	GJQY00000000
Cpac_008		SAMN24144305	SRR17249507	
Chype_012		SAMN24147894	SRR17307982	
Chype_021	*Calanus hyperboreus*	SAMN24147895	SRR17307981	GJRE00000000
Chype_030		SAMN24147896	SRR17307980	
